# Altered thermoregulatory responses in heart failure patients exercising in the heat

**DOI:** 10.14814/phy2.13022

**Published:** 2016-11-15

**Authors:** Bryce N. Balmain, Ollie Jay, Surendran Sabapathy, Danielle Royston, Glenn M. Stewart, Rohan Jayasinghe, Norman R. Morris

**Affiliations:** ^1^ Menzies Health Institute Gold Coast Queensland Australia; ^2^ School of Allied Health Sciences Griffith University Gold Coast Queensland Australia; ^3^ Thermal Ergonomics Laboratory Exercise and Sport Science Faculty of Health Sciences University of Sydney Sydney New South Wales Australia; ^4^ Charles Perkins Centre University of Sydney Sydney New South Wales Australia; ^5^ Division of Cardiovascular Diseases Mayo Clinic Rochester Minnesota; ^6^ Cardiology Gold Coast University Hospital Gold Coast Queensland Australia

**Keywords:** Blood flow, cardiovascular disease, heat production, vascular conductance

## Abstract

Heart failure (HF) patients appear to exhibit impaired thermoregulatory capacity during passive heating, as evidenced by diminished vascular conductance. Although some preliminary studies have described the thermoregulatory response to passive heating in HF, responses during exercise in the heat remain to be described. Therefore, the aim of this study was to compare thermoregulatory responses in HF and controls (CON) during exercise in the heat. Ten HF (NYHA classes I–II) and eight CON were included. Core temperature (*T*_c_), skin temperature (*T*_sk_), and cutaneous vascular conductance (CVC) were assessed at rest and during 1 h of exercise at 60% of maximal oxygen uptake. Metabolic heat production (*H*_prod_) and the evaporative requirements for heat balance (*E*_req_) were also calculated. Whole‐body sweat rate was determined from pre–post nude body mass corrected for fluid intake. While *H*_prod_ (HF: 3.9 ± 0.9; CON: 6.4 ± 1.5 W/kg) and *E*_req_ (HF: 3.3 ± 0.9; CON: 5.6 ± 1.4 W/kg) were lower (*P* < 0.01) for HF compared to CON, both groups demonstrated a similar rise in *T*_c_ (HF: 0.9 ± 0.4; CON: 1.0 ± 0.3°C). Despite this similar rise in *T*_c_, *T*_sk_ (HF: 1.6 ± 0.7; CON: 2.7 ± 1.2°C), and the elevation in CVC (HF: 1.4 ± 1.0; CON: 3.0 ± 1.2 au/mmHg) was lower (*P* < 0.05) in HF compared to CON. Additionally, whole‐body sweat rate (HF: 0.36 ± 0.15; CON: 0.81 ± 0.39 L/h) was lower (*P* = 0.02) in HF compared to CON. Patients with HF appear to be limited in their ability to manage a thermal load and distribute heat content to the body surface (i.e., skin), secondary to impaired circulation to the periphery.

## Introduction

Regular physical activity is endorsed as an integral therapeutic modality for the management of heart failure (HF) (Heart Failure Society of America 2010). whereas most structured and/or supervised exercise‐training programs typically take part in temperature‐controlled environments (Piotrowicz and Wolszakiewics 2008), HF patients may be encouraged to perform physical activity outside of formal rehabilitation programs, which can take place under a range of environmental conditions, including outdoors in a warm environment. A number of reports have shown that environmental heat exposure impacts HF‐associated morbidity and mortality (Semenza et al. 1996; Keatinge 2003; Hausfater et al. 2010), suggesting that thermal tolerance to environmental heat stress may be altered in these patients.

Exposure to increased environmental temperatures causes a number of physiological responses integral for thermoregulation (Rowell 1993). Cutaneous vasodilation and accompanying increases in skin blood flow both serve to distribute heat content to the body shell/surface (i.e., skin) to maintain core temperature within a safe range (Green et al. 2006). The thermoregulatory redistribution of blood flow is a fundamental cardiovascular response (Rowell 1993); however, to offset a decrease in vascular resistance (via cutaneous vasodilation), cardiac output increases in healthy individuals to prevent a catastrophic decrease in blood pressure (Rowell 1993). Although HF patients exhibit impaired myocardial function (Heart Failure Society of America 2010), systemic vasoconstrictor activity is enhanced to compensate for a reduced cardiac reserve to regulate blood pressure (Tei et al. 1995). As such, the autonomic and cardiovascular dysfunction associated with HF may in turn alter the capacity to increase the delivery of blood to the skin during a thermal challenge (Green et al. 2006; Benda et al. 2015a).

Although sweating responses appear to be preserved in HF patients (Cui et al. 2005, 2013), previous studies have shown that HF patients demonstrate attenuated increases in skin blood flow, as evidenced by reductions in cutaneous vascular conductance (CVC) for a given rise in core temperature during passive whole‐body heating (Cui et al. 2005; Green et al. 2006). To date, studies have utilized passive whole‐body heating techniques at supraphysiological temperatures, which does not permit the extrapolation of the findings to realistic whole‐body open air scenarios. In addition, attenuated rises in skin blood flow and skin temperature have previously been reported in HF compared to control (CON) participants during exercise in a thermoneutral environment (Zelis et al. 1969; Benda et al. 2015a). However, the aforementioned studies did not take into account differences in biophysical properties such as body mass and surface area, as well as the management of heat content and the importance of evaporation relative to dry heat loss (convection and radiation) (Gagnon et al. 2013; Cramer and Jay 2014). Importantly, it is now recognized that to perform an unbiased comparison of thermoregulatory responses between independent groups, biophysical properties associated with heat production and mass must be controlled (Jay and Cramer 2015). Indeed, how HF patients manage a thermal load during exercise under environmental heat stress while accounting for these factors remains to be described.

Therefore, the purpose of this study was to examine thermoregulatory responses and human heat balance parameters in HF patients, compared to age‐matched healthy CON participants during exercise in a warm environment, while accounting for differences in body mass and the biophysical properties associated with heat production. It was hypothesized that HF patients would demonstrate an impaired thermoregulatory response primarily through a smaller rise in CVC compared to CON.

## Methods

### Participants

A power calculation (G*Power version 3.1.9.2, Heinrich‐Heine‐Universität Düsseldorf, Düsseldorf, Germany) was performed in order to determine the required sample size for the experiment. Based on conventional *α* (0.05) and *β* (0.95) values, and an effect size of 2.22 as in a previous study (Cramer and Jay 2014) using a similar design (i.e., independent groups) and primary outcome variables (i.e., core temperature), a minimum sample size of 16 participants (8 per group) was required.

A total of 18 men volunteered to participate in this study; 10 HF patients (NYHA classes I–II) who were recruited through the local Community Heart Failure Program of Gold Coast Health Services, and 8 CON recruited from the surrounding community. Patients with HF were eligible to participate on the basis of the following criteria: aged 50–75 years; were within NYHA classes I–II; no recent exacerbation of symptoms relating to HF within the past 3 months with no change in medications; free from implantable devices including a pacemaker and/or defibrillator; and were free from any restriction of ambulation and mobility. CON was eligible to participate if they matched the study population for age, /gender, body mass, and body surface area; were apparently healthy nonsmokers; free from cardiopulmonary, neurological, and/or metabolic diseases and any restriction of ambulation and mobility; and were not taking any cardiovascular medications at the time of participation in the study. Prior to all testing, the study purpose and experimental protocols were disclosed, and all participants provided written and witnessed informed consent. The experimental procedures were reviewed and approved by the Griffith University Human Research Ethics Committee, and complies with the guidelines set out in the Declaration of Helsinki.

### Study design

All participants visited the laboratory on two separate occasions, with each visit separated by at least 48 h. Participants refrained from strenuous physical activity, and consuming food and beverages containing caffeine and/or stimulants for 24 h prior to visiting the laboratory. During the first visit, participants underwent preparticipation health screening, and performed a medically supervised incremental cycling test on a cycle ergometer to determine peak exercise values (heart rate and oxygen uptake). During the second visit, participants performed a prolonged (60‐min) submaximal cycling test in a warm (30°C) laboratory environment.

### Incremental cycling test

Incremental cycling tests were performed on an electronically braked upright cycle ergometer (Lode Corival; Lode BV, Groningen, The Netherlands) for the determination of peak exercise values (oxygen uptake and heart rate). The tests comprised a 3‐min warm‐up period of unloaded cycling, before the workload was increased by 10 W (HF) or 15 W (CON) every 60 s until the participant reached volitional fatigue or symptom limitation. Cardiac rhythm and pulmonary gas exchange were measured via 12‐Lead electrocardiography (ECG) (X12+, Mortara Instrument, Milwaukee, WI) and indirect calorimetry (Ultima, CardiO_2_; Medical Graphics Corporation, St. Paul, MN), respectively. Peak heart rate and oxygen uptake ($\dot{V}O_{2\mathrm{peak}}$ ) were determined as the highest 60 s bin‐averaged values attained during the test.

### Submaximal cycling test

Participants consumed a telemetric temperature sensor capsule (Equivital EQ02; Hidalgo, Cambridge, U.K.) (Byrne and Lim 2012) ~6 h preceding the cycling test. Prior to entering the laboratory, participants were instrumented in a thermoneutral (22°C) environment with a 12‐Lead ECG to monitor cardiac rhythm and measure heart rate, an optic probe (MP1‐V2; Moor Instruments, Milwey, U.K.) on the forearm (which was stabilized to ensure measurement accuracy) ~3 cm distal to the cubital fossa to measure skin blood flux (an index of skin blood flow), and the Equivital system (Equivital EQ02; Hidalgo) to record core (*T*_c_) and skin (*T*_sk_) temperature. Following instrumentation, participants entered the laboratory, which was heated to an ambient temperature of 30°C, and were seated on the upright cycle ergometer for 10 min of quiet rest (baseline). Once the 10‐min baseline period concluded, participants commenced cycling at a preferred cadence without a warm‐up period. The target cycling power output was 60% $\dot{V}O_{2\mathrm{peak}}$ , and participants maintained the workload for 60 min. Immediately following the cycling test, participants were weighed nude so as to determine whole‐body sweat rate. All measurements were monitored continuously, and recorded at baseline and at 10‐min intervals during the cycling test.

Blood pressure was also measured at these time points by manual brachial artery auscultation using a mercury sphygmomanometer (Baumanometer Standby Model; W.C. Baum Co., Copiague, NY).

Pulmonary gas exchange variables were measured, as described for the incremental exercise test, during the final 3 min of the baseline rest period, and at 10 min intervals (3‐min measurement bins) during the submaximal cycling test for the determination of metabolic energy expenditure (*M*) as previously described (Jay et al. 2011):

$$M=\dot{V}O_{2}\frac{\left(\frac{\text{RER}-0.7}{0.3}e_{c}\right)+\left(\frac{1-\text{RER}}{0.3}e_{f}\right)}{60}1000\;\left(W\right)$$

where $\dot{V}O_{2}$ represents pulmonary oxygen uptake, RER represents the respiratory exchange ratio (Jay et al. 2011), and *e*_c_ and *e*_f_ represent the caloric energy equivalent for the oxidation of carbohydrate (i.e., 21.13 kJ) and fat (i.e., 19.62 kJ) per liter of oxygen consumed. To determine metabolic energy expenditure in W/kg, *M* was divided by body mass.

### Heat balance calculations

On the day of testing, subjects were instructed to wear light, loose fitting clothing (i.e., shorts and t‐shirt) so that dry insulation and evaporative resistance of clothing were considered negligible. All parameters were divided by total body mass to derive units in W/kg. Metabolic heat production (*H*_prod_) was determined as the difference between *M* and the external workload (W):

$$H_{\mathrm{prod}}=M-W\;\left(W/\text{kg}\right)$$

The rate of dry heat loss (*H*_dry_) was determined as:

$$H_{\mathrm{dry}}=R+C\;\left(W/\text{kg}\right)$$

$$R=h_{r}\;\cdot \left(T_{\mathrm{sk}}-T_{a}\right)\;\left(W/\text{kg}\right)$$

$$C=h_{c}\cdot \left(T_{\mathrm{sk}}-T_{a}\right)\;\left(W/\text{kg}\right)$$

where *R* and *C* represent radiant and convective heat exchange, respectively; *T*_sk_ and *T*_a_ represent mean skin and ambient temperature (°C), respectively; and *h*_r_ and *h*_c_ are the radiant and convective heat exchange coefficients, respectively (Cramer and Jay 2014):

$$h_{r}=4\cdot 0.77\cdot \varepsilon \cdot \sigma \;{\left[\left(\frac{T_{\mathrm{sk}}+T_{r}}{2}\right)+273.15\right]}^{3}\;\left(W\cdot m^{-2}\cdot K^{-1}\right)$$

$$h_{c}=8.3\cdot v^{0.6}\left(W\cdot m^{-2}\cdot K^{-1}\right)$$

where 0.77 represents the nondimensional effective radiant surface area for a seated individual (Kerslake 1972); *ԑ* represents the emissivity of the skin (0.95); *σ* represents the Stefan–Boltzmann constant (5.67 × 10^−8^ W^**·**^m^−2**·**^K^−1^); *T*_r_ represents the mean radiant temperature, which is assumed to equal *T*_a_ (°C); and *v* represents air velocity in m/s. Respiratory heat exchange (*H*_res_) was determined as:

$$E_{\mathrm{res}}+C_{\mathrm{res}}=\left[0.0014\cdot H_{\mathrm{prod}}\cdot \left(34-T_{a}\right)+0.0173\cdot H_{\mathrm{prod}}\cdot \left(5.87-P_{a}\right)\right]\;\left(\text{W/kg}\right)$$

$$P_{a}=\frac{\text{Relative}\;\text{humidity}\cdot P_{\mathrm{sa}}}{100}\left(kP_{a}\right)$$

$$P_{\mathrm{sa}}=0.1\cdot \text{EXP}\cdot \left[18.956-\left(\frac{4030.18}{\left(T_{\mathrm{sk}}+235\right)}\right)\right]\left(kP_{a}\right)$$

where *E*_res_ and *C*_res_ represents evaporative and convective heat loss from the respiratory tract, respectively; *P*_a_ represents the evaporative and convective ambient vapor pressure (*kP*_a_); and *P*_sa_ represents the saturated vapor pressure (*kP*_a_). The evaporative requirement for heat balance (*E*_req_) was determined as:

$$E_{\mathrm{req}}=H_{\mathrm{prod}}-H_{\mathrm{dry}}-H_{\mathrm{res}}\;\left(\text{W/kg}\right)$$

The calculation of *E*_req_ was based on the 3‐min average values of each heat balance parameter included in the equation. Potential evaporative heat loss from the skin (*E*_sk_) – assuming all secreted sweat evaporated – was determined as (Parsons 2014):

$$E_{\mathrm{sk}}=\frac{\left(\text{WBSR}\cdot 1000\cdot 2426\right)}{3600}\;\left(W\right)$$

where WBSR represents whole‐body sweat rate (calculated based on pre‐ and postexercise changes in nude body weight factoring in intraexercise fluid consumption, which was measured using a dedicated measuring cylinder), which was expressed relative to *E*_req_ to account for differences in *H*_prod_ elicited by the experimental protocol; and the number 2426 represents the heat of vaporization of water in (J/g) (Wenger 1972).

### Thermometry

*T*_c_ and *T*_sk_ (chest, shoulder, anterior thigh, and calf) were monitored continuously using the Equivital system. An area‐weighted mean skin temperature was subsequently calculated, as previously described (Ramanthan 1964):

$$T_{\mathrm{sk}}=0.3\cdot T_{\mathrm{chest}}+0.3\cdot T_{\mathrm{shoulder}}+0.2\cdot T_{\mathrm{thigh}}+0.2\cdot T_{\mathrm{calf}}\;{(}^{\circ }C)$$

To account for the relative contribution of core and skin temperatures to the increases in skin blood flow, mean body temperature (*T*_b_) was subsequently calculated as (Gagnon and Kenny 2012):

$$T_{b}=0.9\cdot T_{c}+0.1\cdot T_{\mathrm{sk}}\;{(}^{\circ }C)$$

Forearm CVC was estimated from skin blood flux divided by mean arterial pressure (Cui et al. 2005). CVC was then expressed as a change from baseline resting values relative to changes in *T*_b_.

### Statistical analysis

Statistical analysis was performed using SPSS 22.0 (SPSS Inc, Chicago, IL). Between‐group (HF and CON) participant characteristics, including peak incremental exercise test, and heat balance data were assessed using paired t‐tests. A two‐way analyses of variance (HF vs. CON) with repeated measures was performed to examine whether changes in hemodynamic and thermometry measurements differed across time, as the within‐participant factor between groups (HF vs. CON). Pair‐wise comparisons using Bonferroni adjustments were applied when a significant main effect was detected. A linear regression analysis was employed to determine the contributions of *T*_b_ to the change in CVC during exercise. Statistical significance was accepted at *P* < 0.05. All data are presented as mean ± standard error of the mean.

## Results

### Participant characteristics

Eighteen men; 10 HF (NYHA classes I–II) and 8 CON were matched for age, gender, and body mass and surface area. All participants completed the incremental exercise test. As expected, HF demonstrated a lower absolute and relative $\dot{V}O_{2\mathrm{peak}}$ compared to CON and consequently, the peak power obtained was lower in HF compared to CON (Table 1). All participants completed the submaximal cycling test in a similar (*P* > 0.05) laboratory temperature (HF: 30.2 ± 0.3; CON 30.5 ± 0.2°C) and relative humidity (HF: 60.0 ± 0.7; CON 58.8 ± 0.8%).

**Table 1:** Participant characteristics

Demographic and functional measures	HF	CON
Age (year)	60 ± 7	62 ± 7
Height (m)	1.76 ± 6.0	1.78 ± 5.1
Body mass (kg)	91 ± 11	82 ± 11
Body mass index (kg/m^2^)	29.3 ± 3.5	26.7 ± 2.4
Body surface area (m^2^)	2.1 ± 0.1	2.0 ± 0.1
Mean arterial pressure (mmHg)	89 ± 5	94 ± 7
Heart rate (beats/min)	67 ± 18	63 ± 14
Peak heart rate (beats/min)	136 ± 19	150 ± 14
$\dot{V}O_{2\mathrm{peak}}$ (L/min)	1.6 ± 0.4	2.5 ± 0.6^a^
$\dot{V}O_{2\mathrm{peak}}$ (ml/kg/min)	18.0 ± 3.5	31.2 ± 9.0^a^
Peak power (W)	97 ± 41	188 ± 60^a^
*Cardiovascular medications*		
ACE inhibitors	8 (80%)	
Beta‐blockers	6 (60%)	
Diuretics	6 (60%)	
Lipid lowering	6(60%)	
Anticoagulants	3 (30%)	

Data are mean ± SD. HF, heart failure participants; CON, control participants; $\dot{V}O_{2\mathrm{peak}}$, peak oxygen uptake; ACE, angiotensin‐converting enzyme.

a Significantly different between HF and CON participants.

### Hemodynamic responses

**Figure 1:**
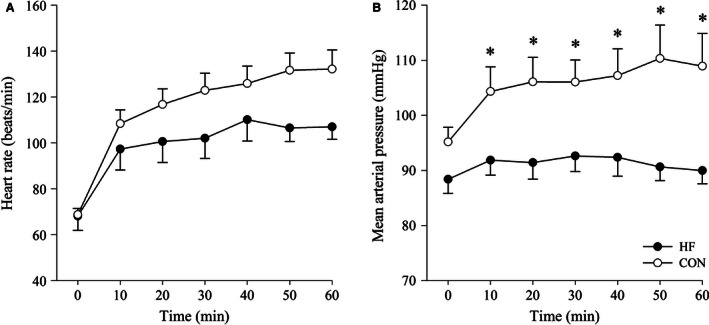
Heart rate (A) and mean arterial pressure (B) for HF and CON participants recorded at 10‐min intervals during exercise. HF, heart failure; CON, control. Data are mean ± SEM. *Significantly different between groups, *P* < 0.05.

Heart rate (Fig. 1A) increased from the onset of exercise in both groups (*P* < 0.01; group × time interaction: *P* = 0.87). Mean arterial pressure (Fig. 1B) increased from the onset of exercise and remained stable thereafter in CON; in HF, mean arterial pressure did not change from rest, and consequently, remained lower (*P* < 0.01) than CON for the duration of exercise.

### Heat balance parameters

The biophysical properties associated with heat production are displayed in Table 2. The group mean external workload was 58 ± 25 W for HF, and 109 ± 33 W for CON (*P* < 0.01). Due to exercising at a lower external workload, absolute *H*_prod_ and *H*_prod_ per unit body mass were lower for HF compared to CON. Similarly, *E*_req_, *H*_res_, and *H*_dry_ were lower in HF compared to CON. Furthermore, the estimated skin surface evaporation from whole‐body sweat losses – assuming complete evaporation – *E*_sk_ was lower for HF than CON; however, the difference in *E*_sk_ relative to *E*_req_ (HF: 84 ± 22; CON: 117 ± 41%) was similar (*P* = 0.38) between the two groups.

**Table 2:** Biophysical properties associated with heat production in both HF and CON during the submaximal cycling test

	HF	CON
*H*_prod_ (W)	356 ± 89	516 ± 114^a^
*H*_prod_ (W/kg)	3.9 ± 0.9	6.4 ± 1.5^a^
*E*_req_ (W/kg)	3.3 ± 0.9	5.6 ± 1.4^a^
*H*_res_ (W/kg)	0.20 ± 0.06	0.32 ± 0.07^a^
*H*_dry_ (W/kg)	0.31 ± 0.05	0.43 ± 0.09^a^
*E*_sk_ (W/kg)	2.6 ± 1.0	6.7 ± 3.2^a^

Data are mean ± SEM. HF, heart failure participants; CON, control participants; *H*_prod_, metabolic heat production; *E*_req_, evaporative requirements for heat balance; *H*_res_, respiratory heat loss; *H*_dry_, dry heat loss; *E*_sk_, evaporative heat potential.

a Significantly different between HF and CON participants.

### Thermometric responses

**Figure 2:**
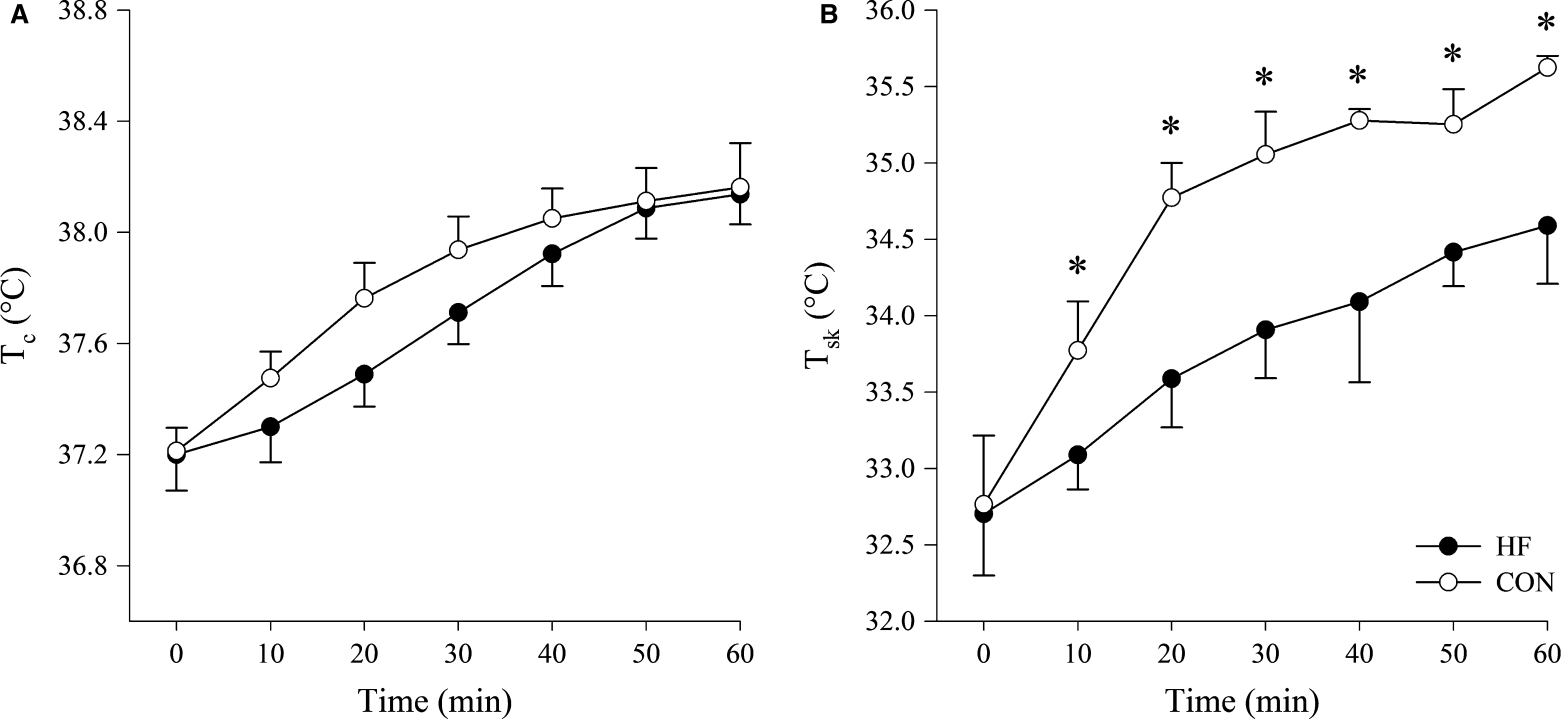
*T*_c_ (A) and *T*_sk_ (B) for HF and CON participants recorded at 10‐min intervals during exercise. *T*_c_, core temperature; *T*_sk_, skin temperature; HF, heart failure; CON, control. Data are mean ± SEM. *Significantly different between groups, *P* < 0.05.

For both groups, there was a similar and significant increase in *T*_c_ (Fig. 2A, *P* < 0.01; group × time interaction: *P* = 0.21); however, *T*_sk_ increased to a greater extent during exercise in CON compared to HF (Fig. 2B, group × time interaction: *P* = 0.04).

### Forearm CVC

**Figure 3:**
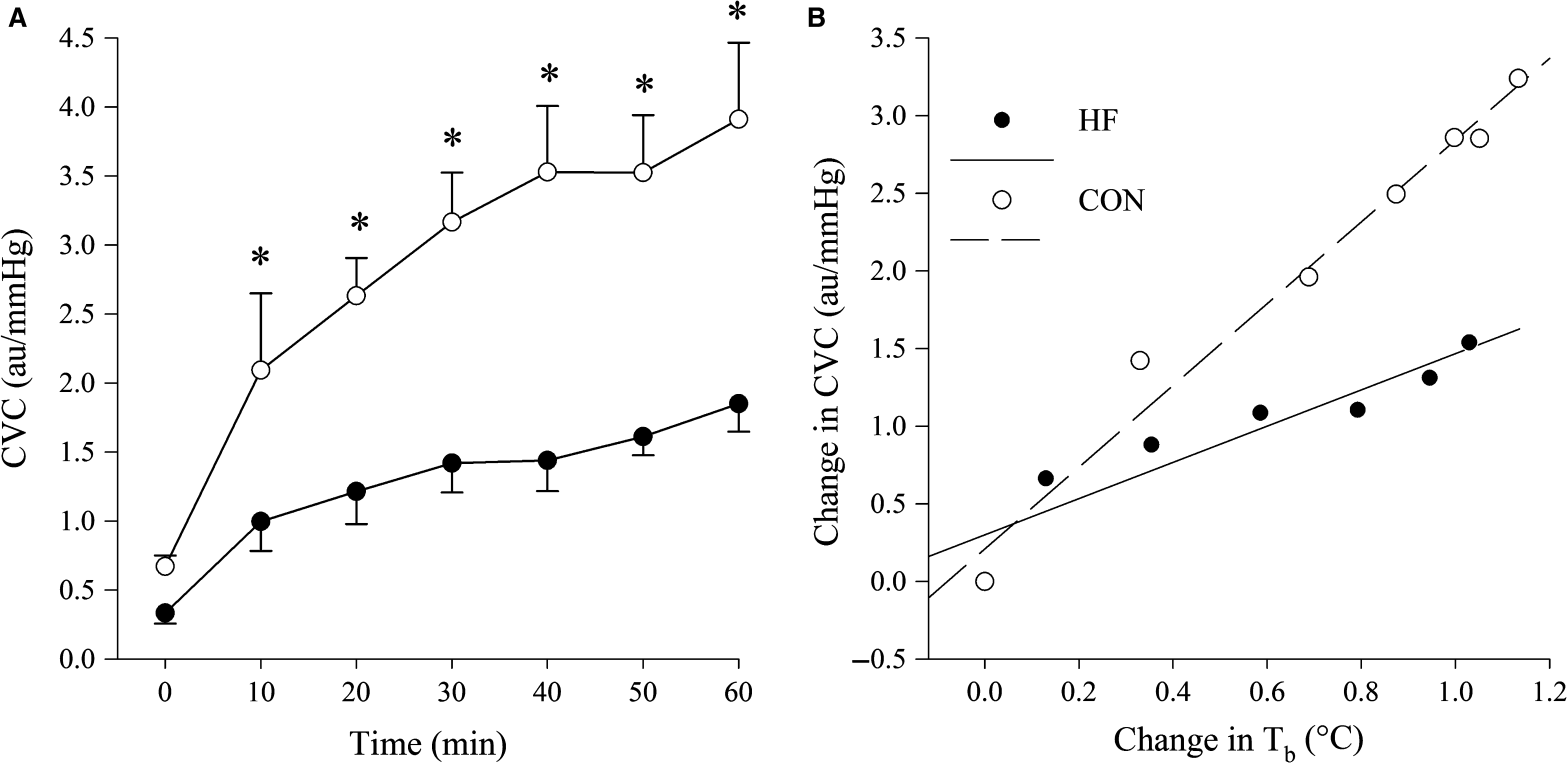
CVC values recorded at 10‐min intervals during exercise (A), and changes in CVC (B) in response to increases in *T*_b_ for HF and CON participants. CVC, forearm cutaneous vascular conductance; *T*_b_, mean body temperature; HF, heart failure; CON, control. Data are mean ± SEM. *Significantly different between groups, *P* < 0.05.

Cutaneous vascular conductance (Fig. 3A) increased to a greater extent during exercise in CON compared to HF (group × time interaction: *P* < 0.01), and the change in CVC relative to the change in *T*_b_ (HF: 1.3 ± 0.9; CON: 2.6 ± 0.9 change in CVC/°C) was lower (*P* = 0.02) in HF compared to CON (Fig. 3B).

### Sweating

Whole‐body sweat rate was lower (*P* = 0.02) in HF (0.36 ± 0.15 L/h) than CON (0.81 ± 0.39 L/h); however, was similar (*P* = 0.83) between groups when corrected for differences in *E*_req_.

## Discussion

This is the first study to examine both the thermoregulatory responses and heat balance parameters in HF during exercise. Our findings show that when exercising in a warm environment, HF had comparable changes in *T*_c_ to CON, even though metabolic heat production per unit mass (i.e., in W/kg) was greater in the latter. This similar *T*_c_ response was accompanied by a blunted rise in CVC in HF, whereas whole‐body sweat rate was similar to CON when accounting for differences in *E*_req_. These findings demonstrate for the first time that thermoregulatory capacity in HF is disrupted during exercise combined with a thermal challenge.

During environmental heat exposure, increases in skin blood flow serve to promote the convective transfer of heat content from the core to the skin. The general notion is that increasing skin blood flow raises skin temperature, thereby widening the skin‐to‐ambient temperature gradient and facilitating heat loss (Benzinger 1969). In this study, although both groups demonstrated similar responses in *T*_c_, *T*_sk_ (and in turn, *H*_dry_) was lower in HF when compared to CON. These findings are similar to that of Benda et al. (2015b) who documented attenuated rises in skin temperature in HF patients compared to healthy controls during cycling exercise in a thermoneutral environment. These authors suggested that lower skin temperatures in HF patients may be reflective of an inability to increase skin blood flow (and raise skin temperature). Consistent with this suggestion is our observation that CVC, and consequently, the transfer of heat content from the body core to the periphery was diminished in HF compared to CON during exercise.

The cutaneous vasculature is under dual sympathetic innervation consisting of an adrenergic vasoconstrictor, and an active cholinergic vasodilatory system which is responsible for most (up to ~95%) of the total rise is skin blood flow (Kellogg 2006; Charkoudian 2010; Johnson 2010). Indeed, a number of studies have demonstrated that peripheral vasoconstriction is enhanced in HF patients due to an overactive sympathetic nervous system (Mancia 1990; Grassi et al. 2003; Watson et al. 2006). Thus, it is likely that the reduced skin blood flow (as evidenced by a diminished CVC) in HF in this study may be, in part, due to enhanced sympathetic neural drive, and/or impaired cutaneous active vasodilator activity at the local level. Apart from neural mechanisms, previous studies have shown that ~30% of the thermoregulatory‐induced elevation in skin blood flow is mediated through NO‐dependant mechanisms (Kellogg et al. 1993, 1998, 1999). Green et al. (2006) demonstrated that NO‐dependant cutaneous vasodilation is impaired in HF patients when compared to healthy controls during passive whole‐body heating. Hence, it may be argued that impaired NO‐dependant cutaneous vasodilation may have at least partially contributed to the reduction in CVC in HF patients in this study.

Exercise coupled with environmental heat stress imposes a significant challenge to the human cardiovascular system (Kenney and Johnson 1992; Rowell 1993). As such, competition may exist for limited cardiac output among blood pressure regulation, active skeletal muscle perfusion, and skin blood flow when this population is exposed to a thermal stress while performing exercise. If increases in cardiac output are not sufficient to offset the combined demands of the skin and active skeletal muscle, then the regulation of blood pressure will be challenged (Wendt et al. 2007). Such a challenge to blood pressure regulation will induce a baroreceptor reflex‐mediated response that has previously been shown to prevent further increases in skin blood flow even in the presence of hyperthermia (Kellogg et al. 1990; Crandall et al. 1996). Consistent with the above, blood pressure was well maintained in HF during exercise in this study, despite being lower than CON.

It is noteworthy that evaporative heat loss from the skin (*E*_sk_) was lower in HF compared to CON; but, when expressed relative to the evaporative requirements for heat balance (*E*_req_), no differences were evident between the two groups. Considering that changes in *T*_c_ were similar in this study, these findings suggest that internal heat management is compromised in HF. As a result, HF patients may have an impaired ability to redistribute internal heat content among various tissues in the body, and that internal heat storage is concentrated more toward the body core (Benda et al. 2015b). The fact that HF demonstrated a lower rise in CVC for a given change in *T*_c_ (i.e., thermosensitivity) compared to CON in this study lends some support to this suggestion.

In addition to skin blood flow, the evaporation of sweat is an effective means of dissipating heat to the environment. Previous studies have documented that *E*_req_ is the primary factor that determines whole‐body sweat rate under conditions permitting complete evaporation (Jay et al. 2011; Gagnon et al. 2013). In this study, absolute whole‐body sweat rate was lower in HF compared to CON. This was expected given that HF were exercising at a lower *H*_prod_, and *E*_req_ represents a simple rearrangement of the heat balance equation by combining *H*_prod_ and the rate of *H*_dry_ (Gagnon et al. 2013). As a result, whole‐body sweat rate when corrected for differences in *E*_req_ was similar between groups. Similarly, previous studies have shown that sweating responses as well as skin sympathetic nerve activity are comparable between HF and CON participants during passive whole‐body heating (Cui et al. 2005, 2013). Collectively, these findings as well as the findings of this study suggest that temperature sensing, and efferent sympathetic cholinergic sweat gland innervation and function may be preserved in HF.

### Methodological considerations

Similar to others, this study compared thermoregulatory responses between HF and CON using a fixed relative exercise intensity (%$\dot{V}O_{2\mathrm{peak}}$) (Tankersley et al. 1991; Inoue et al. 1999; Okazaki et al. 2002; McEntire et al. 2006; Smith and Havenith 2012). The rationale to use such a protocol is based on the original findings of Saltin and Hermansen (1966), which led to the notion that interindividual variability in core temperature regulation during exercise is eliminated when the exercise intensity is expressed as a %$\dot{V}O_{2\mathrm{peak}}$. However, recent evidence suggests that changes in core temperature are primarily determined by *H*_prod_ per unit mass (Gagnon et al. 2009; Jay et al. 2011; Cramer and Jay 2014). Indeed, differences as little as ~1.5 W/kg in *H*_prod_ have been demonstrated to result in significant changes in core temperature between groups with identical physiological control of their thermoregulatory system (Cramer and Jay 2014). However, in this study we observed similar responses in *T*_c_ between the two groups, despite HF exercising with a much lower *H*_prod_ (i.e., ~2.5 W/kg). Hence, our results suggest that the ability to regulate core temperature during exercise is disrupted in HF, either through blunted heat loss responses and/or a less uniform distribution of heat content between the body core and peripheral tissues. Although our findings suggest that HF patients likely exhibit impairments in heat management, we note that future studies should prescribe exercise that elicits a fixed rate of *H*_prod_ in W/kg to assess thermoregulatory capacity by comparing core temperature responses during exercise between experimental (i.e., HF patients) and CON groups (Cramer and Jay 2014).

This study examined HF patients who continued with standard care procedures, which included taking a variety of cardiovascular medications. Thus, it cannot be ruled out that thermoregulatory responses observed during exercise in HF may have been confounded by concurrent use of medication (Pescatello et al. 1987; Lomax and Schönbaum 1998; Chen et al. 2002). Although we acknowledge the confounding influence of cardiovascular medications, we did not attempt to discontinue standard care procedures to allow for the extrapolation of data to the broader populations of HF patients, and daily situations.

## Conclusion

Despite exercising with a lower *H*_prod_ per unit mass, our findings show that skin blood flow responses (i.e., CVC) during exercise in the heat are diminished in HF, whereas sweating responses are not impaired. As such, it appears that HF patients are limited in their ability to manage an endogenous thermal load, secondary to poorer circulation to the periphery. Although routine exercise is recommended for the management of HF, the findings of this study suggests that activity performed at a moderate intensity, coupled with environmental heat stress may predispose individuals with HF to a substantial (~1°) elevation in core temperature. Indeed, this may place HF at a greater risk of potentially larger elevations in core temperature and thus, heat‐related events or a syncopal episode when these patients are exposed to, or are exercising at higher environmental temperatures.

## Conflict of Interest

None declared.
